# Treatment of Acne‐Induced Facial Post‐Inflammatory Hyperpigmentation With a New 2‐MNG‐Containing Routine Versus a Thiamidol‐Containing Routine—A Monocentric Prospective Double‐Blind Randomized Comparative Trial

**DOI:** 10.1111/jocd.70913

**Published:** 2026-06-10

**Authors:** Thierry Passeron, Guénaëlle Le Dantec, Samir Salah, Beatrice Marie, Kate Randamy, Kim T. F. C. Dhin‐Kien, Ann' Laure Demessant‐Flavigny, Andrew Alexis

**Affiliations:** ^1^ Department of Dermatology University Hospital Center of Nice Nice France; ^2^ INSERM U1065 Team 12, C3M Nice France; ^3^ La Roche‐Posay Laboratoire Dermatologique Levallois‐Perret France; ^4^ Insight Research Quatre‐Bornes Mauritius; ^5^ Weill Cornell Medicine New York New York USA

**Keywords:** 2‐MNG, hyperpigmentation, post‐inflammatory hyperpigmentation, skin disorder, stigmatization

## Abstract

**Background:**

Acne‐induced post‐inflammatory hyperpigmentation (PIHP) is a frequent condition. 2‐mercaptonicotinoyl glycine (2‐MNG) is an innovative anti‐pigmenting molecule.

**Aims:**

The aim of this study is to compare the efficacy and tolerability of two routines in the treatment of PIHP: 2‐MNG‐containing serum + SPF50+/UVA sunscreen versus Thiamidol‐containing serum + SPF50+/UVA sunscreen.

**Patients/Methods:**

A prospective, randomized, double‐blind, parallel‐group controlled trial was conducted in Mauritius (February–October 2023). Adults with phototypes III–VI, mild facial acne, and moderate‐to‐severe post‐acne PIHP were recruited. Participants received routine A (2‐MNG‐containing serum+sunscreen) or B (Thiamidol‐containing serum+sunscreen) for 84 days. The primary outcome was the change in PAHPI global score at D84 between routines. Exploratory outcomes included PAHPI global score at earlier time points, as well as PAHPI subscores, lesion darkness, subjective global assessment, color measurements, and stigmatization at D28, D56, and D84.

**Results:**

Eighty‐four patients were screened and included, and 77 were analyzed. At D84, both routines improved the PAHPI global score (*p* < 0.001 vs. D0 for each routine, mean difference between routine A and B at D84: −0.39 ± 1.95, 95% confidence interval [−1.27;0.50], routine A showed no inferiority vs. routine B but did not show superiority), PAHPI lesion number (*p* < 0.01), mean lesion darkness (*p* < 0.001), and stigmatization (*p* < 0.001 for routine A at D56 and for routine B at D84). Routine A was superior to routine B regarding PAHPI lesion number decrease (*p* = 0.03), color measurements at D84 (*p* < 0.05), and satisfaction.

**Conclusions:**

Both routines improved post‐acne PIHP and were well tolerated. The 2‐MNG‐containing routine was not inferior to the Thiamidol‐containing routine and displayed better satisfaction and results on several objective parameters.

## 1 Introduction

Hyperpigmentation disorders, including melasma and post‐inflammatory hyperpigmentation (PIHP), are burdensome skin disorders that cause self‐esteem loss and social stigmatization. PIHP is common, especially in subjects with acne and darker skin phototypes, and affects 58% of acne patients in East Asia, and up to 87% in the Middle East [1, 2]. In women, Perkins et al. found that the highest prevalences for PIHP were in African American and Hispanic women (65% and 48%, respectively) [3]. An older study highlighted a prevalence of acne‐induced PIHP of 65.3% in African American, 52.7% in Hispanic, and 47.4% in Asian patients [4]. This skin condition can either be associated with active acne or persist after acne itself is resolved. It can also occur in the context of traumatization of acne lesions or following aggressive esthetic procedures [5]. It is mainly caused by increased melanogenesis following skin inflammation triggered by endogenous or exogenous factors, and can be worsened by sun exposure [6]. The resultant dark macules are potentially disfiguring, stigmatizing, and are a key dermatologic concern for patients with acne globally [7].

Regarding active acne‐associated PIHP, the gold standard is the acne treatment (i.e., topical retinoids when the lesions are still active) [8], while hydroquinone (HQ)‐based preparations are frequently used in the treatment of post‐acne PIHP [9]. HQ is a tyrosinase inhibitor acting in the very first step of melanogenesis [10, 11]. However, it can induce irritation in the first weeks of application and its long‐term use can result in exogenous ochronosis, which is disfiguring and lacks effective treatment [12]. Other non‐HQ tyrosinase inhibitors have now proven their efficacy in melasma and PIHP, such as Thiamidol‐based preparations [6, 10, 13, 14, 15, 16]. In this article, the Thiamidol routine was thus chosen as the control group because of the published evidence of Thiamidol efficacy in PIHP and other hyperpigmentation conditions [1, 2, 3, 4]. 2‐Mercaptonicotinoyl glycine (2‐MNG, Melasyl) is the newest depigmenting ingredient with a novel mechanism of action, without any action on tyrosinase, conjugating with melanin precursors to inhibit their conversion to eumelanin and pheomelanin pigments [17]. It has demonstrated strong efficacy compared to major depigmenting ingredients in a meta‐analysis of clinical trials [18].

The aim of this study was to test the non‐inferiority of routine A, combining a 2‐MNG‐containing serum with a broad‐spectrum sun protection factor (SPF)50+/ultraviolet A (UVA) sunscreen, versus routine B, which encompassed a Thiamidol‐containing serum combined with a broad spectrum SPF50+/UVA (routine B), during 3 months on the global Post‐Acne Hyperpigmentation Index score (PAHPI, primary outcome), with potential advantages on various clinical objective and subjective parameters at different time points (exploratory outcomes). Improvement was defined as a reduction of at least 1 point in the PAHPI score, which corresponds to the minimally clinically important difference for the PAHPI scale.

## 2 Materials and Methods

### 2.1 Study Design

This study was a monocentric randomized, controlled, double‐blind, parallel‐group study, with before‐after and between‐group comparisons. It was conducted under dermatological control for 3 months in one center in Mauritius from February to October 2023, which corresponds to winter in Mauritius. An independent ethics committee gave its approval on February 23, 2023. Subjects were randomized to either routine A or B based on a simple randomization list with a 1:1 ratio generated by the contract research organization managing the clinical trial. The randomization used permuted block randomization with fixed block sizes of four subjects to allocate participants between the two routines, implemented in Python 3.9.

The two depigmenting routines were as follows: routine A included a 2‐MNG (Melasyl)‐based serum (MelaB3, La Roche‐Posay Laboratoire Dermatologique, France), containing 0.5% 2‐MNG (Melasyl), 10% niacinamide, *Cystoseira Tamariscifolia* extract, lipo‐hydroxy acid, carnosine, retinyl palmitate, dipotassium glycyrrhizate, hyaluronic acid, and glycerin, a combination of active molecules selected to provide greater effectiveness, as well as a broad spectrum SPF50+/UVA sunscreen (Anthelios UVMune 400 SPF50+, La Roche‐Posay Laboratoire Dermatologique, France). Routine B included a serum containing Thiamidol (0.2%), hyaluronic acid, licochalcone A (Eucerin Antipigment Serum Duo, Eucerin, Germany), as well as a broad spectrum SPF50+/UVA sunscreen (Eucerin Oil Control SPF50+, Eucerin, Germany). Thus, our comparisons reflect the overall effects of the two routines, rather than the effects of specific components.

Both routines were provided in a double‐blinded fashion, as both subjects and investigators were blinded to the routine allocation. Products were delivered in neutral encoded packages. Indeed, subjects received the product kits in white tubes that had been repackaged so that the brand or company was no longer recognizable. No blind breaks were reported during the study. Serums had to be applied twice a day on the face and neck in the morning and evening for the 3‐month period. Sunscreens were recommended to be applied twice a day on the face and neck in the morning on top of the serum, with a minimum of 10 min between applications, and in the early afternoon. Both products were to be used for 84 days. The study was conducted in accordance with the good clinical practice, the study protocol, EUROFINS/Insight Research standard operating procedures, and the Declaration of Helsinki [19] and its subsequent amendments.

### 2.2 Patient Population

Healthy adult women and men between the ages of 18 and 50 years with phototypes III to VI were enrolled by the study's investigators. These phototypes were selected because they are more often affected by PIHP than the other skin phototypes. In addition, subjects had to have a mild facial acne at most (Global Acne Evaluation [GEA] = 2) and less than nine inflammatory lesions with moderate to severe PIHP with a Post‐Acne Hyperpigmentation Index (PAHPI) score > 10. They also had to present a maximum of four inflammatory lesions in total. Indeed, active acne severity was strictly controlled at baseline to prevent confounding and clearly differentiate between acne and PIHP [20]. Participants had to provide their free informed written consent, and be willing to adhere to the protocol and study procedures. Patients also gave their consent for the publication of individual patient images.

Non‐inclusion criteria were: subjects with COVID‐19 symptoms, subjects with a temperature above 37.5°C, COVID‐19 positive patients, pregnant or lactating women, women planning to get pregnant during the study, patients with cutaneous pathology on the studied area, use of topical or systemic treatment during the previous weeks (e.g., acne treatment) that might interfere with the cutaneous acceptability/efficacy assessment of the routine, subjects with makeup routine on the visit days, acne and depigmenting treatment (topical or systemic): topical treatment claiming depigmenting effect of the face within the last month, facial procedure within the last 3 months, and systemic retinoid within the last 6 months. Other criteria were the use of cosmetic routine for non‐comedogenicity in the past 2 weeks, subjects treated for PIHP in the past 3 months (dermocosmetic routine or drug), change in hormonal treatment (including contraceptive) in the 3 months before the study, subjects having undergone a surgery under general anesthesia within the previous months, excessive exposure to sunlight or UV‐rays within the previous month, and subjects enrolled in another clinical trial during the study period.

### 2.3 Primary and Exploratory Endpoints

The primary endpoint was the change in the PAHPI global score from baseline at D84 in routine A vs. routine B (comparator routine). Exploratory endpoints were between group comparisons regarding the PAHPI global score at earlier time points as well as comparisons between D0 and each time point for both routines. Other exploratory endpoints included between group comparisons and comparisons vs. D0 within each group for PAHPI subscores (size, intensity, and number of lesions), lesion mean darkness, subjective global assessment (SGA), color measurements, local and global tolerance, stigmatization, and satisfaction at D28, D56, and D84.

### 2.4 Assessments

Patients had to come to the center (Mauritius) for the different visits, during which they were managed by a dermatologist and a technician. At D0, patients were given an information sheet, inclusion/non‐inclusion criteria were checked, and written informed consent was obtained. After this visit, patients applied routine A or B daily and completed a daily diary to record any uncomfortable sensations or medications. Daily logs were also used to assess compliance as patients had to report the number of daily routine applications.

Evaluations were performed at baseline (D0), D28, D56, and D84, including PAHPI global score and subscores (mean darkness, mean size, mean intensity, and number of lesions). Of note, the number of lesions subscore of the PAHPI score refers to the number of PIHP macules (acne marks), rather than active acne lesions. Additional evaluations were subjective PIHP global assessment (SGA, from −1: worsened to 4: completely cleared); mean darkness of PIHP lesions (from 0: normal skin to 8: severe PIHP); standardized clinical pictures were taken with the Colorface system (Newtone Technology) and color measurement was performed using image analysis from the Colorface system (skin lightness [L*] and skin pigmentation [ITA°]); patient‐reported outcomes with stigmatization questionnaire (Patient Unique Stigmatization Holistic in Dermatology [PUSH‐D]: global score, enacted stigma, and felt stigma) [21]; local and global tolerance; and adverse events/treatments were reported. In addition, at D84, satisfaction/cosmetic questionnaires were completed. Dermatological assessments were performed by the same experienced dermatologist, who was accustomed to using the PAHPI scale and performing the abovementioned assessment. The dermatologist was blinded to the routine but not to the visit number.

Regarding colorimetry analyses, calibration was performed as follows: to ensure color consistency: the ColorFace device was calibrated using an automated procedure that uses calibrated test charts, converting raw sensor data into a standardized color space (such as sRGB or Lab*), independent of variations in ambient lighting. For photography, recommendations were to take a photograph of the front face and two profiles with closed eyes, with standard, cross‐polarized, parallel‐polarized, and UV modalities. The hair band should be put the same way each time, the technician should make sure that the subject is not wearing any make‐up or routine, and no hair should be visible to the skin. Face expression should be the same over time (relaxed, no mimic). There should be a valid color chart on all images and images should be of high resolution. Reposition of subjects should be exactly the same at each time point and lighting conditions should remain exactly the same throughout the study.

During the study, the patients had to respect the dates and hours of evaluation visits, remain in the standardized temperature and humidity conditions during study measurements, follow the conditions of use and mode of application of the routines, complete the daily log and bring it back at each visit, avoid excessive UV exposure, and apply the sun routine twice daily even during bad weather. Men had to come clean shaved at each evaluation visit. During the study, the subjects were asked not to apply the routine to test areas on the day of the visits, apply makeup to the test areas on the day of the visit, apply any other similar routine to test areas, modify their makeup/hygiene and/or use new routines, manipulate acne lesions, use acne or hormonal treatment, do professional facial care, use depigmenting and non‐comedogenic skincare and hygiene routine on the face, sunbathe or use tanning routines, or allow the use of the investigational routines by another routine by another person than themselves. Except on visiting days, patients were allowed to use their usual cleansing routines, usual face and eye make‐up removers, and skin care, as long as they did not have depigmenting and non‐comedogenic claims. For women, light face make‐up (powder and blusher), eye and lip makeup were allowed, provided that they did not have depigmenting and non‐comedogenic claims. To be used concomitantly with the study, all cleaning and makeup products were reviewed by the dermatologist.

### 2.5 Statistical Analysis

Subjects who withdrew from the study before the first post‐baseline efficacy evaluation at D28 were excluded from the final analysis. Because these subjects did not contribute to any post‐baseline data, no imputation strategies were applied. The analyzed population thus represents a modified intention‐to‐treat/complete case population, which comprises all randomized subjects who had at least one post‐baseline evaluation.

Descriptive analyses of the data were performed and included means, minimum and maximum values, standard deviations (SD), 95% confidence intervals, percentages of subjects showing improvement, no change, and worsening, as well as variations and percentages of variations. The normality of the differences was determined by the Shapiro–Wilk test, and depending on the result of the normality test, paired Student's *t*‐tests or Wilcoxon signed‐rank tests were performed for intra‐group analyses and unpaired Student's tests or Mann–Whitney tests were carried out for between‐group analyses. A *p*‐value < 0.05 indicated a statistically significant difference. Statistical analyses were performed using Excel and SAS Version 9.4.

The non‐inferiority/superiority hypotheses were pre‐specified in the study protocol before trial initiation and sample size determination was based on the primary endpoint (PAHPI global score) at D84. We established a non‐inferiority margin of 1 point versus routine B, with a hierarchical testing procedure allowing for subsequent superiority testing if non‐inferiority was demonstrated. The 1‐point non‐inferiority margin was grounded in historical data from Tawfic et al. [22], which reported baseline PAHPI scores of 12.32 ± 1.52 and 12.48 ± 1.61 across treatment sides in a comparable post‐acne hyperpigmentation population. These values directly informed our pre‐specified assumptions of a baseline mean of approximately 12 and a standard deviation of 1.56, both confirmed in the current trial. A 1‐point margin represents approximately 10% of this baseline value, reflecting the smallest difference considered clinically meaningful on the PAHPI scale, and remains more conservative than the standard deviation used in the power calculation (SD = 1.56). The calculation used the following parameters, which were defined before data collection: baseline mean PAHPI score of approximately 12, non‐inferiority margin of 1 point, standard deviation of 1.56, type I error rate of 5%, and statistical power of 80%.

## 3 Results

### 3.1 Patient Characteristics

**FIGURE 1:**
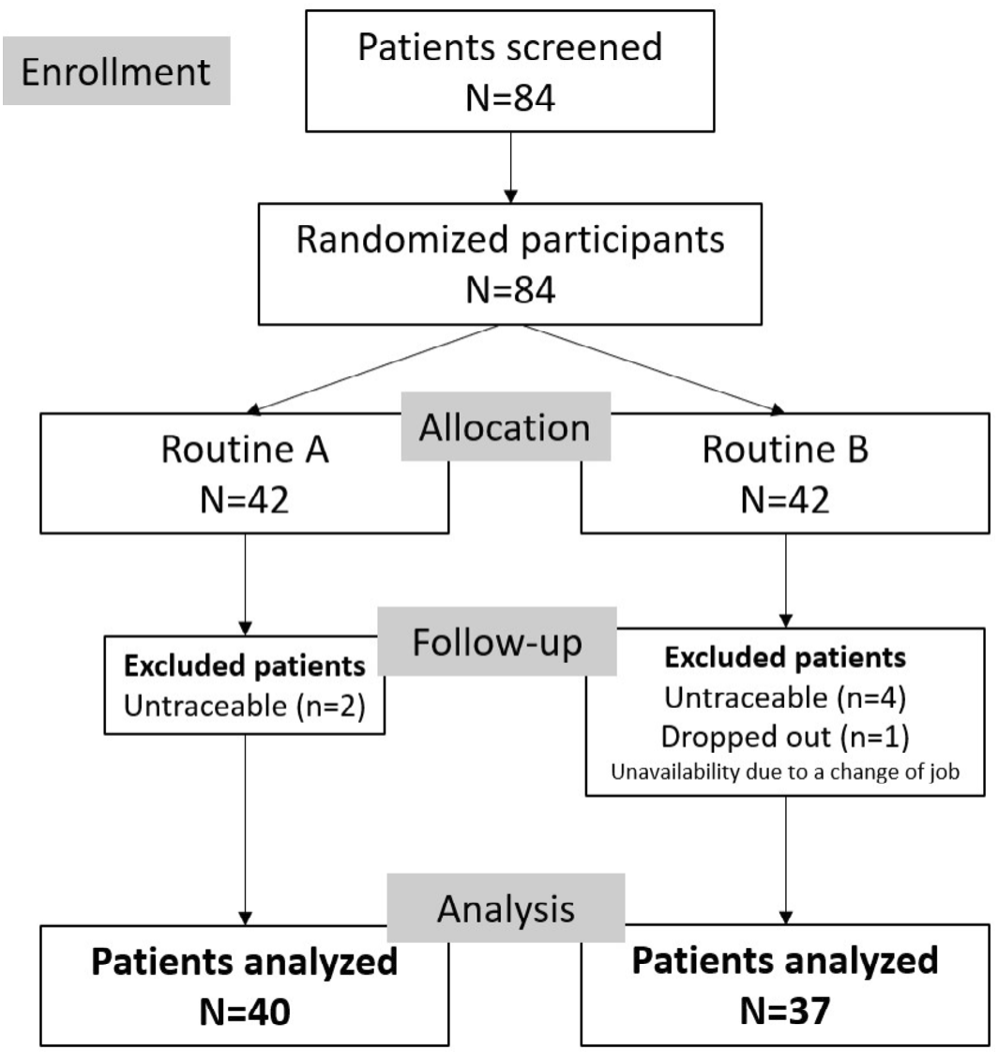
Study flowchart.

Overall, 84 patients were screened, of which 84 were included and 77 were analyzed: 40 for routine A and 37 for routine B (Figure 1). The characteristics of the studied population and reasons for exclusion are presented in Table 1. Most subjects were female (37/40 in routine A and 34/37 in routine B), mean age was 30 years (range: 18 to 50 years old, mean routine A: 31 ± 7 years old, and mean routine B: 30 ± 8 years old), most volunteers had phototype V (25/40 in routine A and 27/37 in routine B), and ethnicity proportions were around one‐third each (African, Indian, and Metis). Mean PAHPI scores at D0 were 12.20 ± 1.54 and 12.03 ± 1.24 for routine A and B, respectively.

**TABLE 1:** Demographic characteristics of the analyzed population (*n* = 77).

Variable	Routine a (*n* = 40)	Routine b (*n* = 37)
Sex, *n* (%)		
*Male*	3 (7.5%)	3 (8.1%)
*Female*	37 (92.5%)	34 (91.9%)
Age, years (mean ± SD)	31 ± 7	30 ± 8
Phototype, *n* (%)		
*IV*	13 (32.5%)	8 (21.6%)
*V*	25 (62.5%)	27 (73%)
*VI*	2 (5%)	2 (5.4%)
Ethnicity, *n* (%)		
*African*	11 (27.5%)	12 (32.4%)
*Indian*	14 (35%)	13 (35.1%)
*Metis*	15 (37.5%)	12 (32.4%)
PAHPI at D0 (mean ± SD)	12.20 ± 1.54	12.03 ± 1.24

Abbreviations: PAHPI, Post‐acne hyperpigmentation index; SD, Standard deviation.

### 3.2 Pigmentation Evaluations: PAHPI Score and Subscores

**FIGURE 2:**
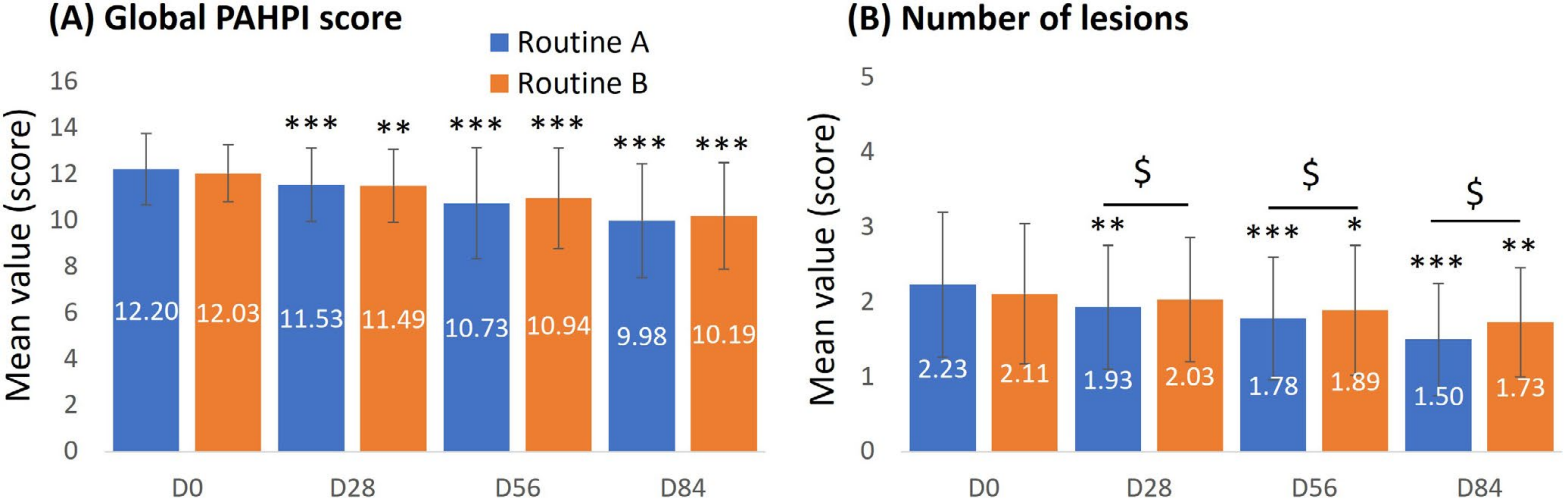
Evolution of the PAHPI global score (A) and lesion number score (B) with routine A and B (mean ± SD). Statistically significant comparisons versus baseline (D0) in each group: *p < 0.05, **p < 0.01, and ***p < 0.001. Statistically significant comparisons between routine A and B0: ^$^ *p* < 0.05. PAHPI: Post‐acne hyperpigmentation index.

The evolution of the PAHPI global score and its subscores is shown in Figure 2. Both routines showed a significant decrease in PAHPI global score compared to D0, with −18% for routine A and −15% for routine B at D84 (*p* < 0.001 versus baseline). Compared to baseline values (D0), this decrease was already significant at D28, indicating an early onset of action, with an improvement in skin appearance. No difference was observed between the two groups at any visit. Regarding the primary outcome, change in PAHPI score at D84 with routine A compared with routine B, non‐inferiority was successfully established between the two treatment groups, but superiority was not significant. With respect to D0, the mean difference between routine A and B was −0.39 ± 1.95 at D84 (95% confidence interval [CI] [−1.27; 0.50]), −0.46 ± 1.63 at D56 (95% CI [−1.22; 0.31]), and −0.13 ± 1.19 at D28 (95% CI [−0.67; 0.41]). Moreover, the upper bound was strictly below the 1‐point non‐inferiority margin. Routine A and B showed similar efficacy on the PAHPI lesion intensity subscore (−20% at D84, *p* < 0.001 in both groups, difference between groups not significant). Routine A had a faster (significant from D28 for routine A vs. from D56 for routine B) and greater effect than routine B on the PAHPI lesion number subscore (−33% vs. −18% at D84, *p* = 0.035), this difference being significant across all visits (*p* = 0.03 also at D28 and D56).

The percentage of subjects showing global PAHPI score improvement was higher with routine A (77.5%) vs. 59.5% with routine B at D84. This difference was consistent over time (40.0% vs. 27.0% at D28 and 67.6% vs. 42.9% at D56 for routine A and B, respectively).

**FIGURE 3:**
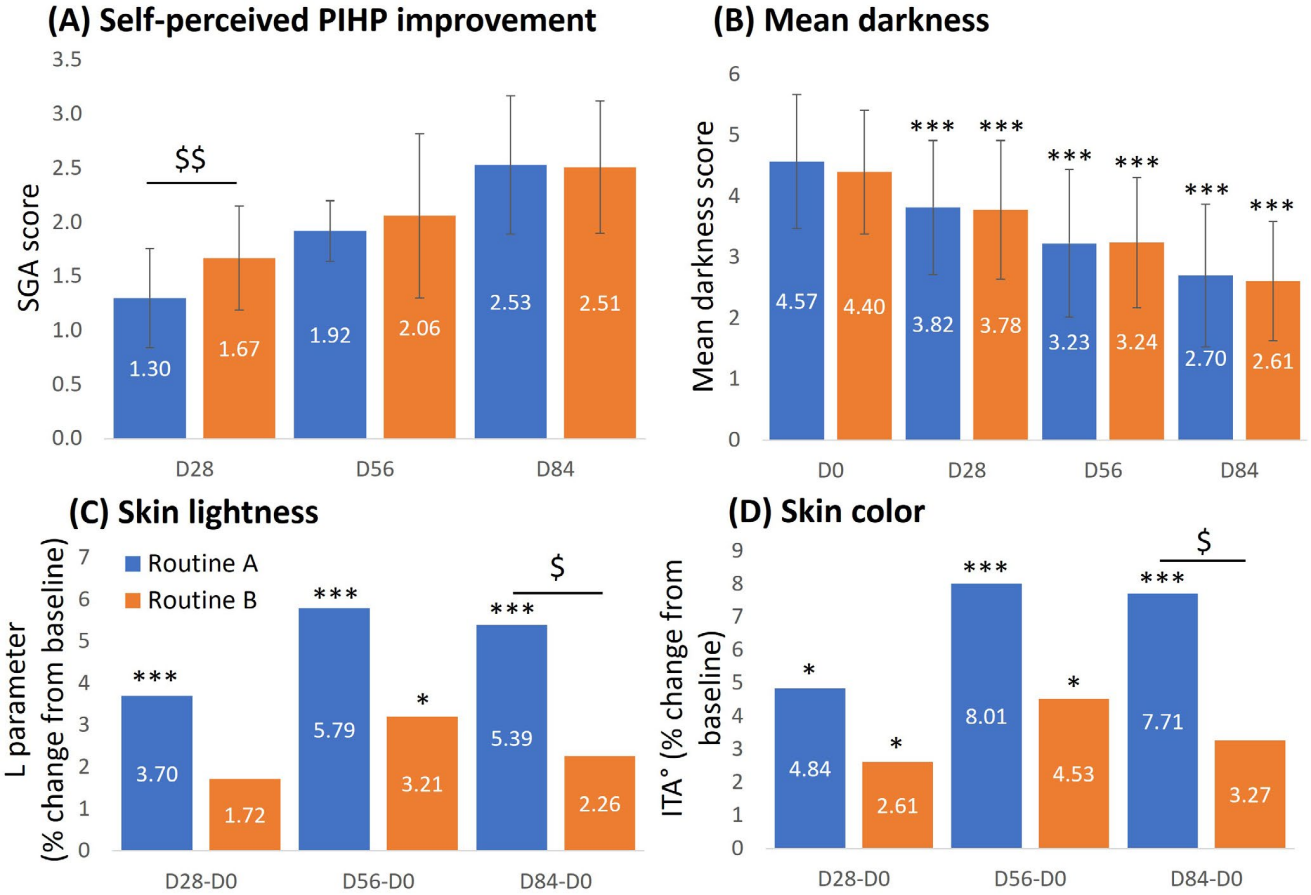
Evolution of self‐perceived PIHP improvement assessed via the SGA score (A), mean darkness clinical evaluation (B), skin lightness (C), and skin color (D) for routine A and B (mean ± SD). SGA: Subjective global assessment, PIHP: Post‐inflammatory hyperpigmentation. Statistically significant comparisons between routine A and B: ^$^ *p* < 0.05, ^$$^ *p* < 0.01. Statistically significant comparisons with baseline (D0) in each group: *p < 0.05, **p < 0.01, and ***p < 0.001.

Self‐perceived improvement in PIHP, as assessed via the SGA score, highlighted an improvement of approximately 2.5 at D84 in both groups, meaning an average of moderate to marked improvement. The SGA score was significantly lower with routine A at D28 compared with routine B (1.30 ± 0.46 vs. 1.67 ± 0.48 at D28, *p* < 0.01, Figure 3A). Of note, 60% (24/40) and 56.76% (21/37) of subjects using routine A and B, respectively, felt markedly improved results at D84, reflecting improvement in PIHP lesions.

### 3.3 Mean Darkness

The results were confirmed by the mean darkness clinical evaluation presented in Figure 3B, which showed a similar significant decrease in both groups (−41% at D84 in both groups, *p* < 0.001), with a significant effect already visible at D28 (−16% for routine A vs. −14% for routine B, *p* < 0.001 in both groups).

### 3.4 Colorface Analysis

**FIGURE 4:**
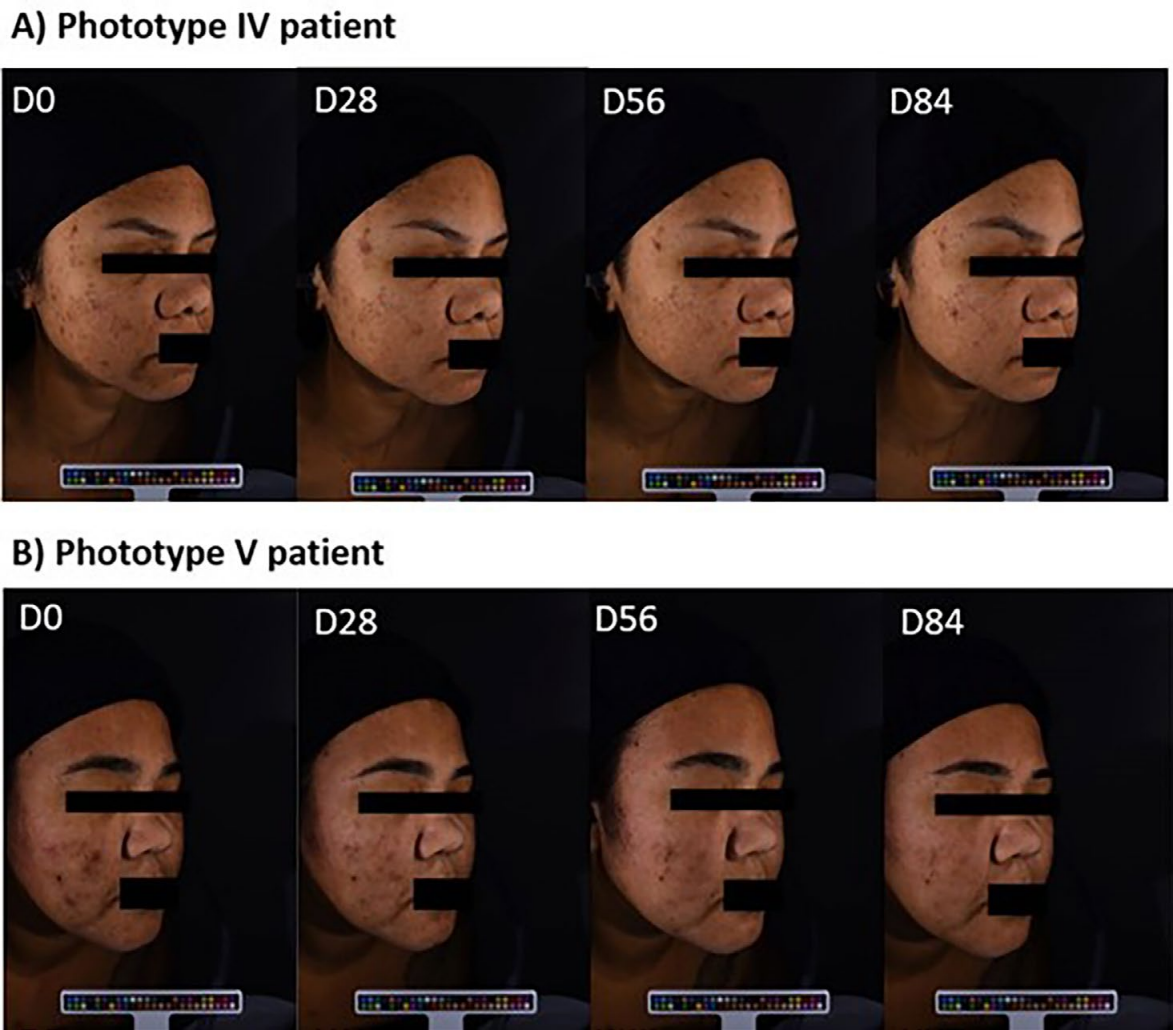
Post‐acne post‐inflammatory hyperpigmentation. Pictures of the Colorface evaluation at D0, D28, D56, and D84 (panels left to right) for two anonymized subjects with phototype IV (A) and phototype V (B).

Regarding color and homogeneity analyses, skin lightness measured by the L* parameter was improved with routine A versus baseline starting at D28 (*p* < 0.001), whereas with routine B skin lightness was significantly improved at D56 versus baseline (*p* < 0.05). At D84, skin lightness improvement was significantly stronger with routine A compared with routine B (*p* < 0.05). Skin color measured by the ITA° parameter was improved at all time points versus baseline for routine A and at D28 and D56 for routine B, with a significantly stronger improvement of skin color at D84 with routine A compared with routine B (*p* < 0.05). The evolution of skin lightness and color is shown in (Figure 3C,D). Pictures of the Colorface evaluation at the different time points in two patients are shown in Figure 4.

### 3.5 Quality of Life

Regarding stigmatization, decreases of −22%, −27%, and −24% in the global PUSH‐D score were observed versus D0 at D28 (−4.60 ± 9.83, *p* < 0.01), D56 (−6.46 ± 14.51, *p* < 0.001), and D84 (−4.98 ± 17.37, not significant) for routine A, respectively. For routine B, a significant decrease was present in the global PUSH‐D score at all time points compared to baseline: −24%, −37%, and −53% at D28 (−5.50 ± 14.67, *p* < 0.05), D56 (−7.77 ± 18.53, *p* < 0.05), and D84 (−11.46 ± 14.70, *p* < 0.001), respectively. The enacted stigma subscore was decreased at D28, D56, and D84 (−13%, −15%, and −7%, respectively) for routine A, although the differences vs. D0 were not significant. For routine B, enacted stigma decreased at all time points (−14% at D28, −15% at D56, and −47% at D84). The difference vs. D0 was only significant at D84 (*p* < 0.05). The felt stigma subscore was also decreased at all time points (−28% at D28, −35% at D56, and −35% at D84, all *p* < 0.01) for routine A. For routine B, felt stigma presented a decrease at all time points (−29% at D28, −47% at D56, and −56% at D84), with all differences vs. D0 being significant (*p* < 0.001). No differences were found between routines over time regarding the global score, as well as the enacted stigma and felt stigma subscores.

### 3.6 Satisfaction and Cosmetic Questionnaires

Overall, 93% of patients found routine A more effective than their usual product, compared to 75% for routine B. Both routines showed very good cosmetic acceptability, as indicated by the rating of many cosmetic criteria. According to the self‐assessment efficacy questionnaire, while both routines had very similar efficacy on dark spots, skin comfort, and hydration, routine A showed slightly better results on fine lines, wrinkles, pores, and skin texture.

### 3.7 Tolerance

No clinical, functional, or physical signs and no serious adverse events or adverse reactions were observed by the investigator at D84 in the two groups. No paradoxical hyperpigmentation was observed.

## 4 Discussion

In this study, we compared a routine including a 2‐MNG‐containing serum and an SPF50+/UVA sunscreen versus a routine including a Thiamidol‐containing serum and an SPF50+/UVA sunscreen, which showed effectiveness on post‐acne PIHP over 3 months of use. Non‐inferiority of routine A versus routine B was demonstrated on the PAHPI global score at D84. We reported a gradual decrease over time in post‐acne hyperpigmentation, number of lesions, mean darkness, and stigmatization with both routines, as well as improvements regarding skin lightness and color. Interestingly, although both enacted and felt stigma subscores decreased versus D0 in both groups, the decrease in the global score was more broadly due to the decrease in the felt stigma subscore, as its decrease was significant at all time points with both routines, which was not the case for enacted stigma. This suggests that the effect of the routines was greater on the patient's feeling of embarrassment linked with PIHP than on the perceived act of discrimination against patients with PIHP [21]. The decrease in stigmatization is likely a consequence of the improvements regarding PAHPI and color analysis parameters. The evolutions were consistent between dermatologist assessments (PAHPI and mean darkness) and subject self‐assessments. However, some additional benefits were observed with the 2‐MNG‐containing routine on exploratory clinical and instrumental endpoints. The improvement of the PAHPI lesion number subscore was faster with routine A, as a significant improvement was visible from D28 with this routine versus from D56 with routine B, and the decrease with routine A was significantly greater than with routine B at D28, D56, and D84. Moreover, skin lightness and skin color presented improvements that were significantly higher with routine A compared with routine B at D84. The improvement in skin lightness versus D0 also occurred earlier with routine A (significant difference starting at D28) compared with routine B (significant difference only at D56). Subjects' self‐perceived improvement demonstrated a clinically relevant effect of both routines, with significantly better results with routine A at D28 compared with routine B. Patient satisfaction was higher with routine A than with routine B. None of the subjects reported any adverse events. Both routines were perfectly tolerated and very well accepted from a cosmetic standpoint.

We must also highlight that as both the serums and sunscreens differed in vehicle, active components, and likely sensory properties, our results reflect the effects of each routine as a whole, rather than the effects of a specific component. Indeed, even though 2‐MNG was the key ingredient, the 2‐MNG serum contained other active ingredients in order to provide better efficacy and answer to the complex pathophysiology of PIHP: lipohydroxy acid and retinyl palmitate, which promote exfoliation and improve skin texture [23, 24], niacinamide and K2G, which exhibit anti‐inflammatory properties, thereby reducing skin irritation and redness [25, 26], and *Cystoseira tamariscifolia* extract, which provides anti‐aging, antioxidant, and anti‐inflammatory properties [26]. Likewise, the Thiamidol‐containing serum contained various components in addition to Thiamidol. Two different sunscreens were included in the two routines. Indeed, current UV filters are now part of pigmentary disorder management and using a broad‐spectrum tinted sunscreen is mandatory in PIHP [27, 28]. Both sunscreens contained UVA and UVB filters, as well as other components, which may have contributed to the effects observed. For instance, the sunscreen in routine A contained tocopherol (vitamin E), which is among the recommended components of sunscreens and anti‐hyperpigmentation dermocosmetics in acne‐induced PIHP prevention due to its antioxidant nature [29]. The sunscreen used in routine B contained licochalcone A (anti‐inflammatory effect), glycyrrhetinic acid (anti‐inflammatory effect), and L‐carnitine (decreased sebum production) [27, 28]. Moreover, we cannot exclude that oil control and finish components might also have influenced the results. Additionally, regarding the self‐ratings of satisfaction and texture/pores, we must mention that the vehicle aesthetics' alone may influence self‐ratings independently of pigment change.

To date, one randomized controlled clinical trial has evaluated the effect of 2‐MNG on UV‐induced darkening and melanin synthesis. This study showed that 2‐MNG inhibits skin pigmentation mechanisms by trapping melanin precursors and new melanin production [18]. In addition, a meta‐analysis on the anti‐pigmenting effect of depigmenting agents compared the efficacy of 2‐MNG against several reference molecules for UV daylight‐induced pigmentation disorders. The authors highlighted the rapid and sustained efficacy of 2‐MNG, with an immediate and long‐lasting effect compared to 13 reference molecules [30]. A first open label study on a Melasyl‐containing serum as a stand‐alone treatment in PIHP patients demonstrated its clinical and instrumental efficacy [26]. Significant decreases in PAHPI score, mean darkness, and spot color intensity were observed at the assessment point (D84). A brightening effect was observed, given the increased ITA° of spots and adjacent areas. Moreover, self‐perceived improvements were reported regarding appearance and well‐being. High product tolerability and cosmetic acceptability of the formulation were also demonstrated in the previous publications [18, 26].

This study has some limitations. First, the number of patients was relatively limited and further research should be carried out with a larger sample size. Second, the monocentric nature of our study may hinder result generalizability and warrants the need for multicentric studies. Third, we must mention the lack of multiplicity control for exploratory endpoints, which was not pre‐specified in the protocol. Fourthly, the proportion of men was 8%, making them strongly underrepresented. Although pigmentary disorders are usually more common in women, men usually represent a sizable proportion of the patient population [31]. Lastly, future trials should include a split‐face design with the same sunscreen and matched vehicles, in order to more cleanly isolate the effects of the serum. Future studies should also explore the effect of the 2‐MNG‐containing routine in lighter phototypes, as we focused on phototypes III‐VI in this study.

As dermatology moves toward artificial intelligence (AI)‐enabled image analytics, and because our outcomes rely on standardized photography and colorimetric endpoints, we must point out the future contribution of AI to dermatology. Multiple groups have shown that AI can support dermatologic workflows through automated lesion detection/segmentation, lesion counting, and severity grading, improving objectivity and reproducibility in acne‐related and other skin condition assessments [32, 33]. In the context of post‐acne PIHP, an AI‐assisted pipeline could (i) automatically segment and count hyperpigmented macules, (ii) quantify area and darkness over time and translate this into a PAHPI‐like digital score, and (iii) harmonize measurements across visits/centers (e.g., device/lighting normalization). This latter point would directly address our limitations regarding monocentric design and scalability.

Overall, by including an action on melanogenesis via a completely new MoA, the 2‐MNG‐containing routine demonstrated good efficacy, equivalent to that of the tyrosinase inhibitor (Thiamidol), as the 2‐MNG‐containing routine led to similar evolutions across time points and proved non‐inferior to the Thiamidol‐containing routine regarding the PAHPI global score improvement. Regarding exploratory endpoints, the 2‐MNG‐containing routine resulted in improvements compared with the Thiamidol‐containing routine on the lesion number, self‐perceived PIHP efficacy, skin lightness, and skin color, with greater patient satisfaction. Therefore, this novel non‐hydroquinone 2‐MNG‐containing routine appears as an effective and well tolerated new approach for the care of acne‐induced PIHP and may be considered a valuable addition to the range of therapies available for patients suffering from post‐acne PIHP.

## Author Contributions

Guénaëlle Le Dantec: Conceptualization, design and conduct of clinical study, data curation; Thierry Passeron and Andrew Alexis: Methodology of clinical study, supervision, manuscript review; Samir Salah: Statistics; Beatrice Marie and Kate Randamy: Conduct of the study; Kim T.F.C. Dhin‐Kien: Clinical study investigator; Ann’Laure Demessant‐Flavigny: Writing, supervision.

## Funding

This work was supported by La Roche Posay Laboratoire Dermatologique.

## Ethics Statement

The study has been approved by an independent ethics committee on February 23, 2023.

## Consent

Consent for the publication of recognizable patient photographs or other identifiable material was obtained by the authors and included at the time of article submission to the journal stating that all patients gave consent with the understanding that this information may be publicly available.

## Conflicts of Interest

T. Passeron has received honoraria and/or consulting fees from ACM, Almirall, AbbVie, Amgen, Astellas, Beiersdorf, Bristol Myers Squibb, Caudalie, Celgene, Galderma, GlaxoSmithKline, Hyphen, Incyte, ISDIN, ISIS Pharma, Janssen, La Roche Posay, LEO Pharma, Lilly, L'Oréal, Merck Sharpe & Dohme, NAOS, Novartis, Pierre Fabre, Pfizer, Sanofi‐Genzyme, Sun Pharmaceutical Industries, SVR, Symrise, Takeda, UCB, Vichy, and VYNE Therapeutics. He is the cofounder of Nikaia Pharmaceuticals. G. Le Dantec, S. Salah, and A.L. Demessant‐Flavigny are L'Oreal employees.

## Data Availability

The data that support the findings of this study are available from the corresponding author upon reasonable request.
